# Posterior ankle arthroscopy for posterior ankle synovitis with an enlarged posterior talar process caused by a cat bite or scratch: A case report

**DOI:** 10.1016/j.ijscr.2021.105761

**Published:** 2021-03-13

**Authors:** Ichiro Tonogai, Koichi Sairyo

**Affiliations:** Department of Orthopedics, Institute of Biomedical Science, Tokushima University Graduate School, 3-18-15 Kuramoto, Tokushima City, Tokushima, 770-8503, Japan

**Keywords:** Cat, Bite, Scratch, Enlarged posterior talar process, Posterior ankle arthroscopy

## Abstract

**Introduction:**

Legs are sometimes infected and swollen by cat bite or scratch. However, there is no report of synovitis with an enlarged talar posterior process in the posterior ankle caused by a cat bite or scratch which was treated by removal of the enlarged process and synovectomy with release of the flexor hallucis longus tendon via posterior ankle arthroscopy.

**Presentation of case:**

The patient was a 58-year-old woman who had started keeping a cat 7 months earlier. She subsequently sustained cat bite and scratch wounds to her lower legs, which gradually became increasingly swollen. On presentation, there was left lower leg swelling, particularly on the posterior aspect of the ankle. Imaging revealed bone marrow edema in the enlarged posterior talar process and inflammation of the adjacent soft tissue. We excised the enlarged posterior talar process, performed synovectomy, and released the flexor hallucis longus tendon using a posterior arthroscopic technique via standard posterolateral and posteromedial portals. Microbial culture was negative.

**Discussion:**

The patient returned to daily activities approximately 3 weeks after arthroscopic debridement. There was no recurrence at the 1-year follow-up visit. To our knowledge.

**Conclusion:**

We report a rare case of posterior ankle synovitis with an enlarged posterior talar process caused by a cat bite or scratch which was treated successfully by posterior ankle arthroscopic debridement.

## Highlights

• Posterior ankle synovitis with enlarged posterior talar process was caused by cat bite/scratch.
• Infected and swollen ankle was treated successfully by posterior arthroscopic debridement.
• There were many advantages in posterior ankle arthroscopy, compared to open ankle surgery.

## 1 Introduction

Cat bites represent 3%–15% of all animal bites [[1], [2], [3], [4]] and cause local infection in 30%–50% of cases [5]. Infected cat bites commonly present as cellulitis but severe infection with tenosynovitis, abscess, arthritis, or osteomyelitis may also occur. In one report, 48% of patients who were hospitalized for an infected cat bite developed complications [6]. Moreover, although polyarthritis is a rare manifestation of cat scratch disease, there has been only one report of bilateral ankle arthritis [7].

The posterior talar process is comprised of medial and lateral tubercles that serve as the respective attachments for the posterior talotibial and talofibular ligaments. A secondary ossification center forms on the posterolateral aspect of the talus between the ages of 7 and 13 years, usually fuses within 1 year, and articulates with the talus via a synchondrosis [[8], [9], [10]]. In 7%–14% of adults, this center remains as a separate accessory bone [11]. If abnormal structures are present, such as an enlarged, prominent, elongated, or hypertrophic posterior talar process (known as a trigonal or Stieda process), the surrounding soft tissue becomes impinged between the posterior distal surface of the tibia and the superior surface of the calcaneus [12]. To our knowledge, there have been no reports of synovitis with an enlarged posterior talar process in the posterior ankle caused by a cat bite or scratch wound.

Here we report a rare case of posterior ankle synovitis with an enlarged posterior talar process caused by a cat bite or scratch which was treated by resection of the enlarged posterior talar process, synovectomy, and release of the flexor hallucis longus (FHL) tendon via posterior ankle arthroscopy. This has been reported in line with the SCARE criteria [13].

## 2 Presentation of case

Informed consent was obtained from the patient for this report to be published.

**Fig. 1:**
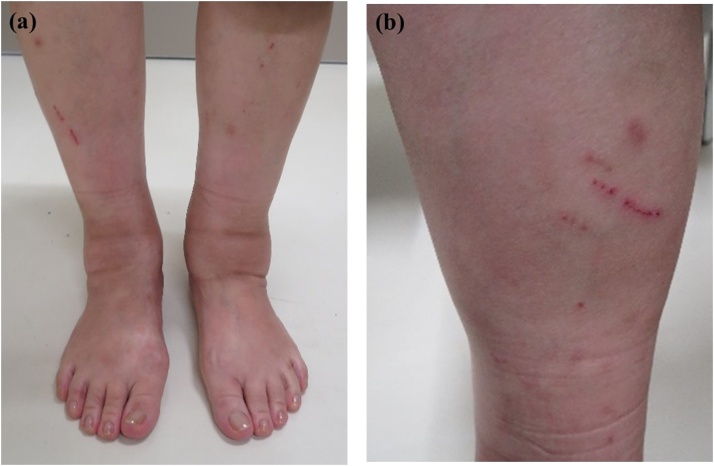
Preoperative photograph showing swelling of the left lower leg on an anterior view (a) and a cat bite or scratch wound on a posterior view (b).

The patient was a 58-year-old woman who was referred to our department with an approximately 5-month history of slight pain on loading and swelling of the left ankle without obvious trauma. Seven months before presentation, her family had started keeping a cat, which often bit and scratched her on the lower legs. Her left lower leg swelling had started 5 months earlier. She was referred to our hospital by a local doctor when her symptoms did not resolve. She had no past medical history. She also had no family history of relevant genetic information and psychosocial history. At her first visit to our department, she complained of muscle fatigue in the left lower leg. Physical examination revealed slight tenderness of the medial and lateral posterior aspects of the ankle and severe swelling of the left lower leg (Fig. 1a, b). There was slightly limited range of motion of the left ankle. She felt posterior ankle pain during plantar flexion on the left side. No neurovascular deficit was noted. There was no axillary lymphadenitis. Synovial fluid culture from the left posterior ankle was negative. Laboratory investigations revealed mild inflammation (white blood cell count, 8700 cells/μL [reference range, 4300–5600]) and a high C-reactive protein level (1.83 mg/dL [reference range, 0.00–0.14]). Her JSSF (Japanese Society for Surgery of the Foot) scale score was 79/100 (pain 30/40, function 39/50, alignment 10/10).

**Fig. 2:**
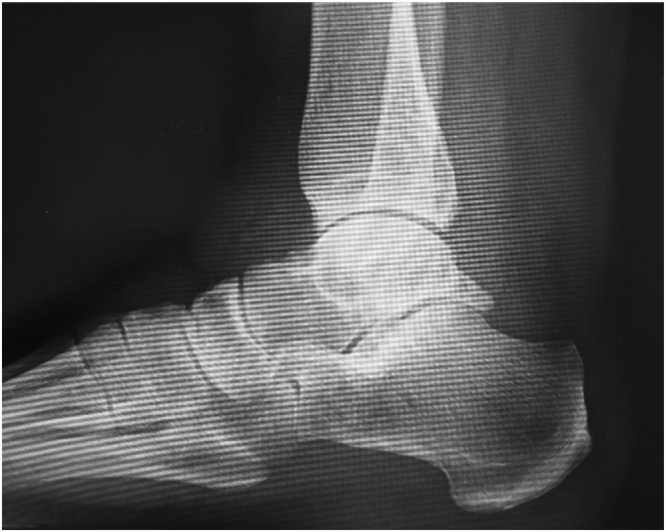
Lateral plain radiographic view of the left ankle obtained preoperatively showing the enlarged posterior talar process in the posterior ankle.

**Fig. 3:**
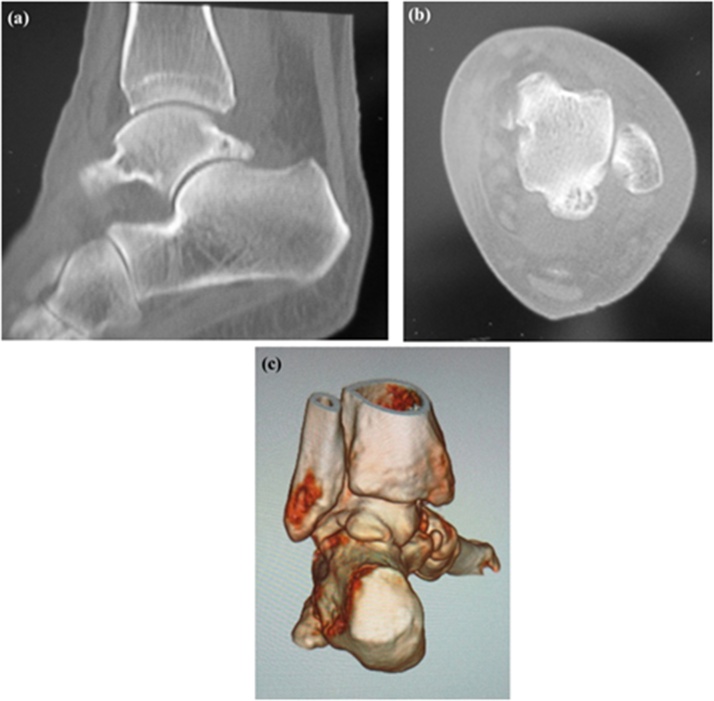
Computed tomography scans showing an enlarged posterior talar process in the posterior ankle (a) on plain sagittal, (b) axial, and (c) three-dimensional views.

**Fig. 4:**
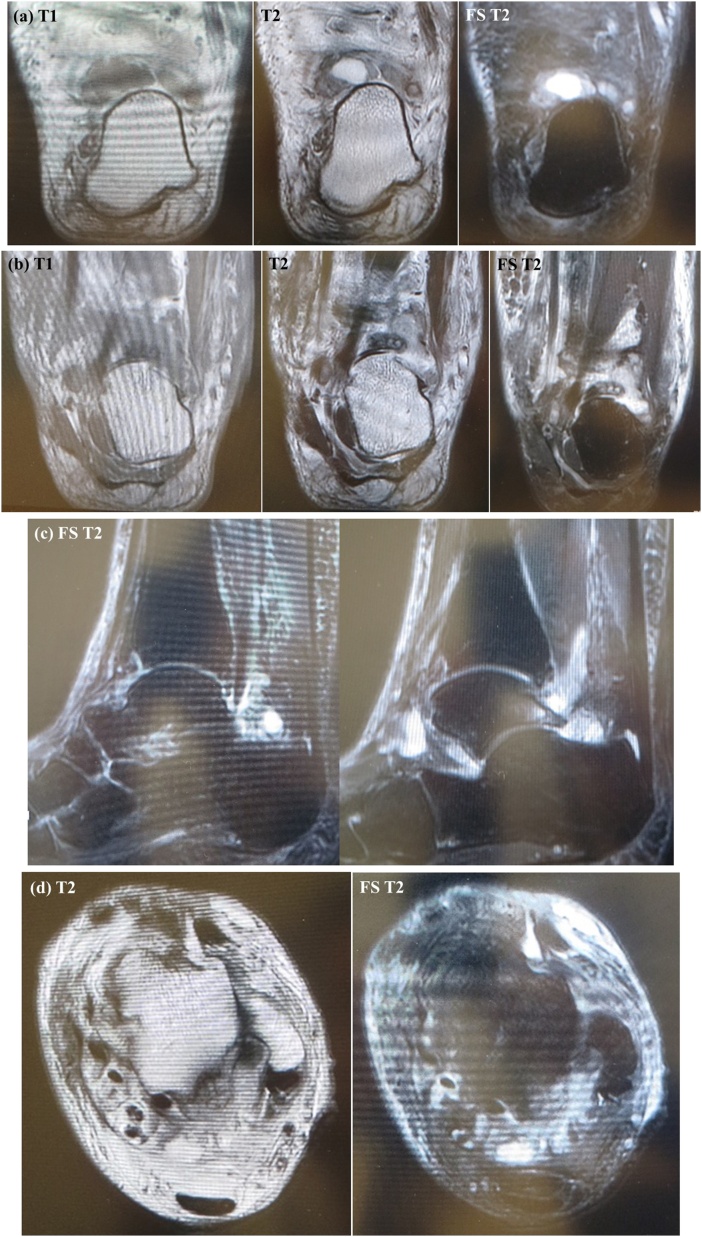
T1-weighted, T2-weighted, and FS T2-weighted magnetic resonance scans in the coronal plane (a, b), an FS T2-weighted scan in the sagittal plane (c), and T2-weighted and FS T2-weighted axial images (d) showing cysts and adjacent soft tissue edema-like signal intensity and bone marrow edema in the enlarged posterior talar process with synovitis in the posterior ankle.

A weight-bearing lateral plain radiographic view of the posterior ankle revealed an enlarged posterior talar process (Fig. 2), which was also seen on computed tomography scans (Fig. 3a–c). Cystic masses and bone marrow edema-like signal intensity in the enlarged posterior talar process with an adjacent soft tissue edema-like signal were seen in the posterior ankle on T1-weighted, T2-weighted, and fat-suppressed T2-weighted magnetic resonance images in the coronal (Fig. 4a, b), sagittal (Fig. 4c), and transverse (Fig. 4d) planes. The preoperative diagnosis was infectious synovitis of the posterior ankle with an enlarged posterior talar process caused by a cat bite or scratch. A plan was made to reduce the patient’s swelling surgically using a minimally invasive arthroscopic approach. The surgery was performed by I.T. who graduated from the medical university in 2004 and was a foot and ankle surgeon.

**Fig. 5:**
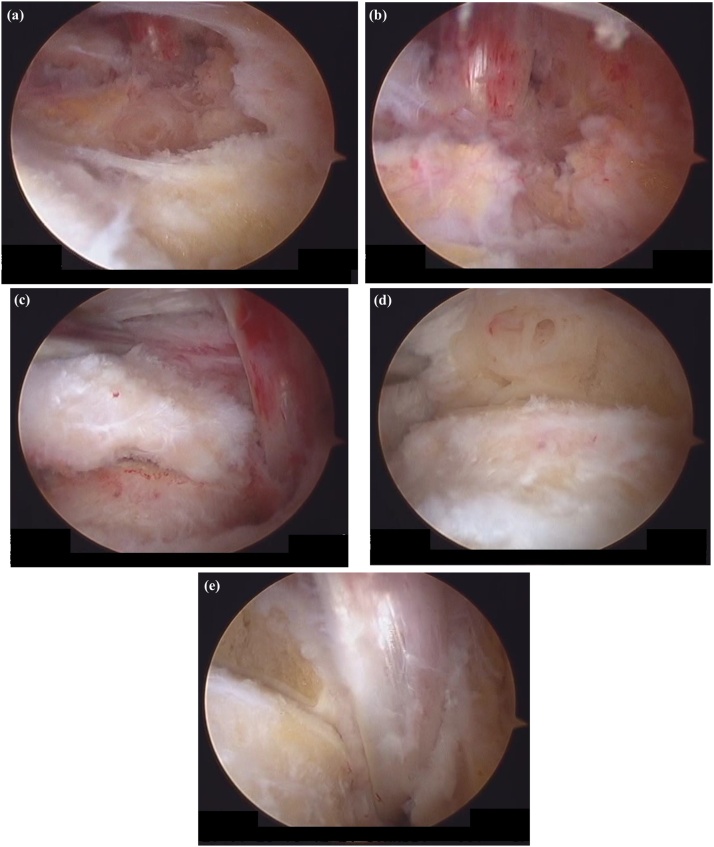
Arthroscopic views of the posterior aspect of the left ankle showing severe synovitis within the posterior ankle and tenosynovitis in the flexor hallucis longus (a, b). The enlarged posterior talar process is seen to be overhanging with friction between the enlarged posterior talar process and the flexor hallucis longus tendon (c). The enlarged posterior talar process and inflamed synovium were removed (d). After aggressive debridement and release of FHL, the FHL moved smoothly (e).

**Fig. 6:**
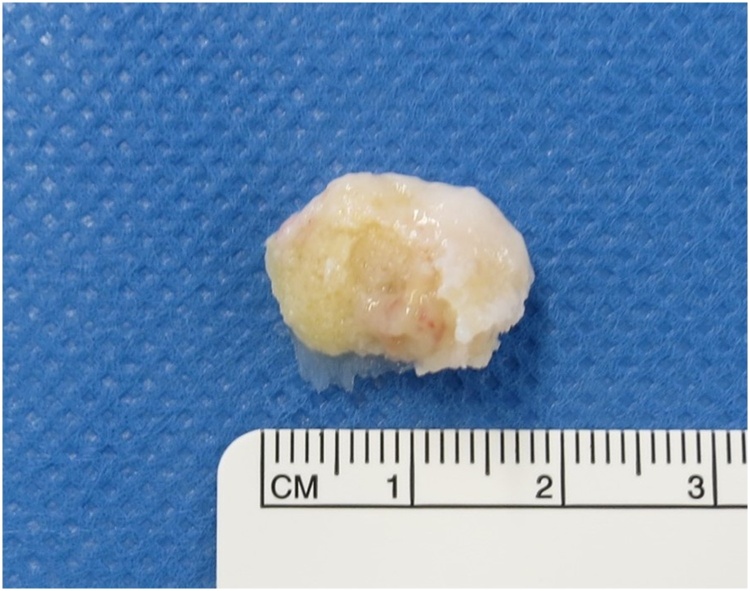
Photograph of the resected enlarged posterior talar process (18 mm in width, 9 mm in length).

**Fig. 7:**
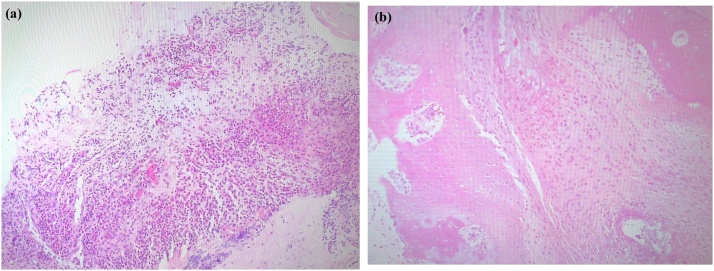
Histopathologic photographs of a sample of synovial tissue (a) and the enlarged posterior talar process (b). (a) Hematoxylin-eosin staining showed chronic synovitis with infiltration of neutrophils and lymphocytes, angiogenesis, and accumulation of hemosiderin, indicating a chronic inflammatory response to infection. (b) Hematoxylin-eosin staining shows infiltration of neutrophils and lymphocytes, hemosiderin, and ingrowth of small new vessels, indicating a chronic inflammatory reaction.

The procedure included irrigation, extensive debridement, and synovectomy with removal of the enlarged posterior talar process via standard anteromedial and anterolateral portals. The patient was positioned prone and a thigh tourniquet was placed. Two portals were created 1 cm above the insertion of the Achilles tendon, one just medial and one just lateral to the tendon, in line with the tip of the lateral malleolus using the standard two-portal technique described by van Dijk et al. [14] The lateral portal was used for visualization and the medial one was used as the working portal. A 4-mm, 30-degree arthroscope was introduced through the portals and directed towards the second toe. Intraoperatively, dense fibrous tissue and aggressive hypertrophic synovitis was seen in the posterior ankle (Fig. 5a, b). There was marked friction between the enlarged posterior talar process and the FHL tendon (Fig. 5c). After endoscopic resection of the hypertrophic posterior process of the talus in the posterior ankle, the severe synovitis and inflamed FHL in the posterior ankle were removed (Fig. 5d). A sample of synovial tissue was obtained for culture and pathological examination. The entire FHL tendon sheath could be visualized and was released down to the entrance of the fibro-osseous tunnel using a shaver (Fig. 5e). We confirmed that the FHL moved smoothly with motion of the great toe. There were no intraoperative complications. The resected enlarged posterior process was 18 mm wide and 9 mm in length (Fig. 6). Histological examination indicated chronic synovitis with infiltration of neutrophils and lymphocytes, angiogenesis, and accumulation of hemosiderin, suggesting chronic inflammation with an infectious etiology (Fig. 7a). Histological findings were consistent with infectious synovitis but synovial tissue culture was negative for organisms such as *Pasteurella multocida* or *Bartonella henselae*. Histological examination of the resected enlarged posterior talar process also indicated chronic inflammation with infiltration of neutrophils and lymphocytes and accumulation of hemosiderin (Fig. 7b).

**Fig. 8:**
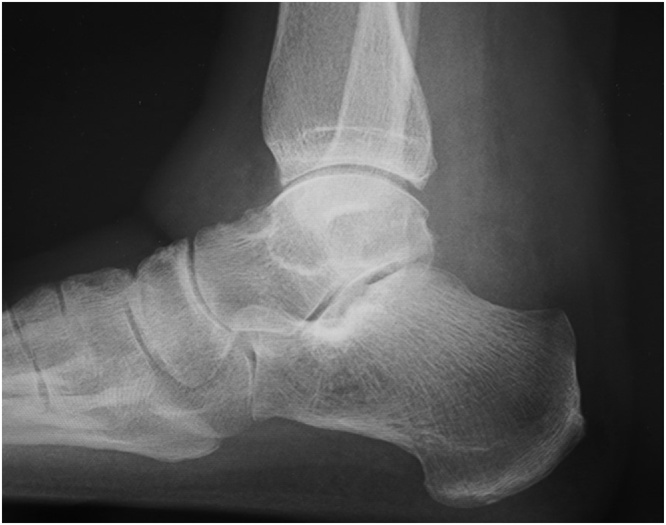
Postoperative plain radiograph acquired after surgery confirms successful resection of the enlarged posterior talar process.

**Fig. 9:**
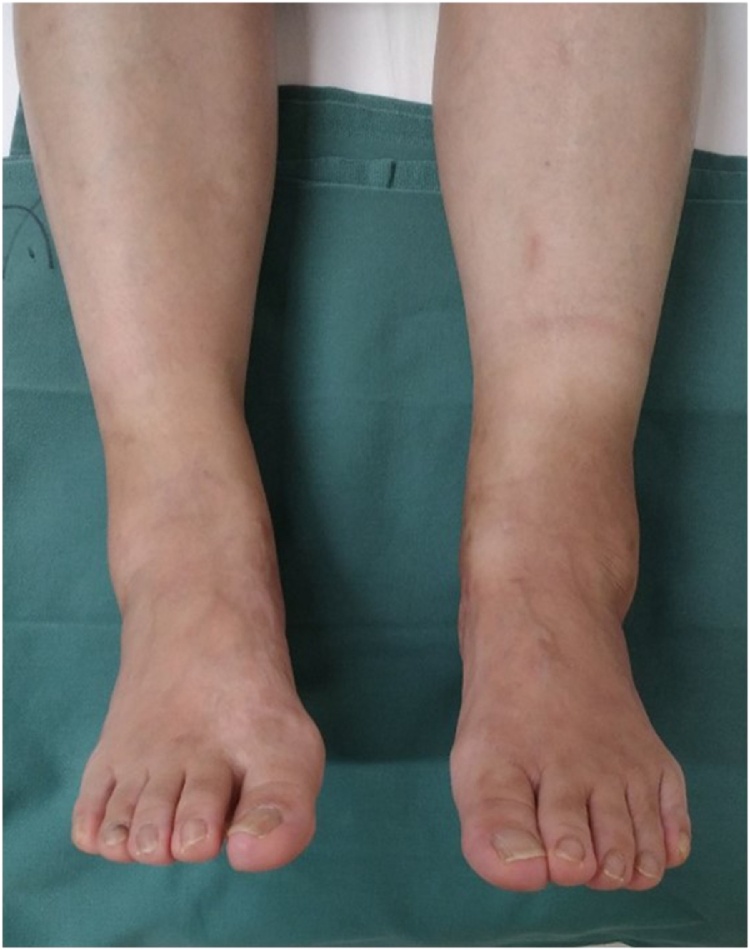
Photograph obtained 2 months after surgery confirms a marked decrease in swelling of the left lower leg.

A bulky dressing was placed postoperatively without immobilization. A lateral plain radiograph confirmed that the enlarged posterior talar process was resected successfully (Fig. 8). The patient was encouraged to actively move her ankle and toes. Weight bearing was allowed after surgery as tolerated, and she was allowed to return to daily activities after 3 weeks. The postoperative course was unremarkable. Her left lower leg swelling had decreased by 2 months after surgery (Fig. 9), at which time her white blood cell count and C-reactive protein level had decreased to 4800 cells/μL and 0.04 mg/dL, respectively.

At the 1-year follow-up visit, the patient was very satisfied with her surgery and reported no limitation of daily activity. Her JSSF scale score had improved from 79/100 to 97/100 (pain 40/40, function 47/50, alignment 10/10).

## 3 Discussion

We encountered a rare case of posterior ankle synovitis with an enlarged posterior talar process in a patient with a history of cat bite and scratch wounds to the lower legs who was treated successfully by posterior ankle arthroscopic debridement

*P. multocida* is a common causative pathogen in human infections from cat or dog bite. A small gram-negative coccobacillus, Pasteurella has been isolated in up to 80%–90% of feline gingival tissue samples (where *P. multocida* predominates) [15,16]. However, it was not cultured in this case. Westling et al. recommended local treatment for an infected cat bite, including drainage and debridement of the wound and irrigation of the affected tendon sheath or joint [6]. Therefore, in this case, we performed debridement in the posterior ankle because we strongly suspected infectious synovitis caused by a cat bite or scratch.

Posterior ankle endoscopy is a useful tool when treating various pathologies of the posterior ankle [14]. It is a minimally invasive surgical procedure that allows good visualization of the involved structures and yields good results [17]. Endoscopic removal of the posterior talar process and synovectomy has the advantages of fewer wound complications, thorough assessment of the posterior ankle, and access to the posterior recesses at this site [18]. Therefore, we selected a posterior ankle arthroscopic approach rather than open surgery to avoid the need for extensive soft tissue dissection. In this case, the outcome was excellent and the patient returned to work within 3 weeks.

A Stieda process can be seen in 14%–25% of normal ankle radiographs [19]; therefore, its presence does not in itself imply posterior ankle impingement syndrome. The posterior talar process could be enlarged in a patient who has posterior ankle impingement syndrome without an os trigonum12 and may be compressed during extreme plantar flexion. Thus, the presence of an enlarged posterior talar process in itself is not sufficient to produce the syndrome. In our patient, the synovitis adjacent to the enlarged posterior talar process might have been worsened by the cat bite or scratch.

In a report by Frigg et al., 13.3% of patients (4/30 feet) developed a painful stress reaction in the posterior subtalar joint after arthroscopic resection of an os trigonum or posterior talar process [20] such that the uncovered calcaneal joint surface was significantly longer in feet that sustained permanent damage than in feet that did not (6.4 mm vs 1.06 mm). They called this the Brisk configuration (the radius of the talus ending within the subtalar joint). Fortunately, in this case, there was no persistent inability to engage in daily activities due to a painful stress reaction in the posterior subtalar joint. However, as recommended by Frigg et al., patients should be informed about the possible risk of a Brisk configuration, although a posterior ankle arthroscopic approach is useful for avoiding this pathology.

The differential diagnosis in this case included cat scratch disease, which was first described in 1950 by Debre and Mollaret [21]. The majority of reported cases have been in persons under 20 years of age, who are usually male [22,23]. However, our patient was a 58-year-old woman. The typical clinical manifestations of cat scratch disease are skin changes at the inoculation site and benign lymphadenopathy. These features were not consistent with this case because lymphadenopathy was absent. The causative agent of cat scratch disease, *B. henselae* [24], was not isolated in this case. Therefore, we ruled out a diagnosis of cat scratch disease.

This report has some limitations. One was the short follow-up duration. Although there was no recurrence of left lower leg swelling due to posterior ankle synovitis at the most recent follow-up visit 1 year after surgery, further follow-up is necessary. Another limitation is that we could not obtain a positive culture from the sample of synovial tissue taken from the posterior ankle. However, we believe that posterior arthroscopic debridement was appropriate based on the diagnosis of infectious posterior ankle synovitis.

## 4 Conclusion

We have encountered a rare case of posterior ankle synovitis with an enlarged posterior talar process caused by a cat bite or scratch. The patient was treated by resection of the enlarged posterior talar process, synovectomy, and release of the FHL tendon via posterior ankle arthroscopy.

## Declaration of Competing Interest

The authors report no declarations of interest.

## Funding

This research received no specific grant from any funding agency in the public, commercial, or not-for-profit sectors.

## Ethical approval

A clinical case report is exempt from ethical approval in our institution.

## Consent

A written informed consent was obtained from the patient for publication of this case report and accompanying images. A copy of the written consent is available for review by the Editor-in-Chief of this journal on request.

## Author contribution

IT was responsible for this study, and managed this study. IT performed surgery. Dr. KS supervised this study. All authors read and approved the final manuscript.

## Registration of research studies

Not applicable.

## Guarantor

Ichiro Tonogai.

Koichi Sairyo.

## Provenance and peer review

Not commissioned, externally peer-reviewed.
